# Location of the CD8 T Cell Epitope within the Antigenic Precursor Determines Immunogenicity and Protection against the *Toxoplasma gondii* Parasite

**DOI:** 10.1371/journal.ppat.1003449

**Published:** 2013-06-20

**Authors:** Virginie Feliu, Virginie Vasseur, Harshita S. Grover, H. Hamlet Chu, Mark J. Brown, Jeremy Wang, Jon P. Boyle, Ellen A. Robey, Nilabh Shastri, Nicolas Blanchard

**Affiliations:** 1 INSERM, U1043, Toulouse, France; 2 CNRS, U5282, Toulouse, France; 3 Université de Toulouse, UPS, Centre de Physiopathologie de Toulouse Purpan (CPTP), Toulouse, France; 4 Division of Immunology and Pathogenesis, Department of Molecular and Cell Biology, University of California, Berkeley, Berkeley, California, United States of America; 5 Department of Biological Sciences, University of Pittsburgh, Pittsburgh, Pennsylvania, United States of America; Cornell University, United States of America

## Abstract

CD8 T cells protect the host from disease caused by intracellular pathogens, such as the *Toxoplasma gondii* (*T. gondii*) protozoan parasite. Despite the complexity of the *T. gondii* proteome, CD8 T cell responses are restricted to only a small number of peptide epitopes derived from a limited set of antigenic precursors. This phenomenon is known as immunodominance and is key to effective vaccine design. However, the mechanisms that determine the immunogenicity and immunodominance hierarchy of parasite antigens are not well understood.

Here, using genetically modified parasites, we show that parasite burden is controlled by the immunodominant GRA6-specific CD8 T cell response but not by responses to the subdominant GRA4- and ROP7-derived epitopes. Remarkably, optimal processing and immunodominance were determined by the location of the peptide epitope at the C-terminus of the GRA6 antigenic precursor. In contrast, immunodominance could not be explained by the peptide affinity for the MHC I molecule or the frequency of T cell precursors in the naive animals. Our results reveal the molecular requirements for optimal presentation of an intracellular parasite antigen and for eliciting protective CD8 T cells.

## Author Summary

*Toxoplasma gondii* is a widespread intracellular parasite that can cause severe disease in immunocompromised individuals and lead to fetal abnormalities if contracted during pregnancy. Establishment of protective immunity relies on CD8 T cells, which recognize antigenic peptides presented by MHC class I molecules on the surface of *T. gondii*-infected cells. Intriguingly, while the proteome of *T. gondii* is large, CD8 T cell responses target a very limited set of peptides. These peptides can be ranked according to the magnitude of the associated CD8 response (from immunodominant down to subdominant). Yet, little is known about the rules that define their immunogenicity and the hierarchy of the associated T cell responses. Using a panel of genetically modified *T. gondii* where the GRA6 dominant antigen was mutated, we show that the C-terminal location of the epitope within the source antigen is the critical parameter for immunodominance. Interestingly, when placed at the C-terminus of GRA6, the subdominant status of an epitope can be overturned. Our results unravel the mechanisms that make parasite antigens accessible for the MHC I presentation pathway. They may help to ameliorate natural immune responses and improve vaccine design against intravacuolar pathogens.

## Introduction

CD8 T cells play a critical role in immune-mediated protection against intracellular apicomplexan parasites. Antigenic determinants recognized by CD8 T cells are short peptides of 8 to 10 amino acids presented by class I molecules of the major histocompatibility complex (MHC I). Antigenic peptides are typically degraded by cytosolic proteasomes, transported into the endoplasmic reticulum (ER), trimmed by ER-resident aminopeptidases and loaded on peptide-receptive MHC I molecules [1]. The spectrum of peptides that can theoretically be presented by a given MHC I is far larger than the peptides that actually elicit CD8 T cell responses. Furthermore, not all the peptide-MHC I complexes that can be recognized are equal: rather they elicit a hierarchy of specific CD8 T cells. This phenomenon of “selection and ranking” is termed immunodominance. Immunodominant peptide-MHC I elicit the most abundant cognate T cell populations, whereas subdominant peptide-MHC I induce less abundant T cells (reviewed in [2], [3]). Knowledge of the mechanisms that enhance immunogenicity and determine immunodominance hierarchy is central to design of optimal vaccines.

Mechanisms of immunodominance have been widely studied in the context of viral infections. The dominant position in the hierarchy has been positively correlated with 1) efficiency of peptide generation by the antigen processing pathway, e.g. due to proteasomal activity [4], ER aminopeptidase activity [5] or the nature of epitope-flanking sequences [6]), 2) antigen abundance [7], 3) ability of the antigen-presenting cells (APCs) to stimulate T cells, e.g. dendritic cells (DCs) *versus* non-professional APCs [8], 4) MHC binding affinity [4], [9] and 5) size of the naïve pool of specific T cells [9], [10], [11]. This latter parameter is increasingly being considered as a good predictor of immunodominance hierarchy, although, like the other parameters, it does not seem to be absolute [12].

During infection by intracellular parasites, the parameters that promote immunogenicity of a protein and that determine T cell immunodominance remain largely unknown. Unlike viruses, parasite-derived antigens are not synthesized by the host cell translation machinery, thus bypassing a preferential linkage between protein synthesis and MHC I presentation [13]. Moreover, except for antigens that may be directly injected into the host cytoplasm (e.g. *T. gondii* rhoptry proteins), most antigens from parasites that live in vacuoles are segregated from the cytosol by one or more membranes. We hypothesize that, despite the greater genomic complexity of apicomplexan parasites relative to viruses [14], these key differences could determine the limited number of hitherto characterized antigenic peptides from *Plasmodium yoelii* [15] *Theileria annulata* [16] or *T. gondii* [17] parasites.

In the present study, we addressed the causes and consequences of immunodominance during *T. gondii* infection. *T. gondii* is a widespread intravacuolar parasite that can cause severe disease in humans [18]. *T. gondii* replicates in a specialized parasitophorous vacuole (PV) and CD8 T cells play a protective role, especially against toxoplasmic encephalitis which is caused by the persistence of cysts in the brain [19]. We previously identified a decamer peptide (HF10, derived from the GRA6 protein) presented by the L^d^ MHC I molecule and recognized by a large CD8 T cell population during toxoplasmosis [17]. Two other epitopes, also presented by L^d^, have been reported: the ROP7-derived IF9 and the GRA4-derived SM9 peptides [20]. Although the source antigens for each of these epitopes are contained in *T. gondii* secretory organelles, the GRA6-specific response appeared immunodominant based on its magnitude [17]. The molecular mechanisms for the potent immunogenicity of GRA6-derived HF10 epitope are not known.

We generated transgenic parasites that do or do not express the GRA6-derived HF10 epitope and established that even in the absence of the immunodominant GRA6-specific CD8 T cell response, the subdominant GRA4 and ROP7 responses remain poorly immunogenic and fail to protect mice from toxoplasmosis. We show that the location of the epitope at the C-terminus of the GRA6 antigenic precursor is a critical parameter that allows efficient processing, determines immunodominance and provides protection during chronic stage.

## Results

### A transgenic model to investigate parasite immunogenicity

**Figure 1:**
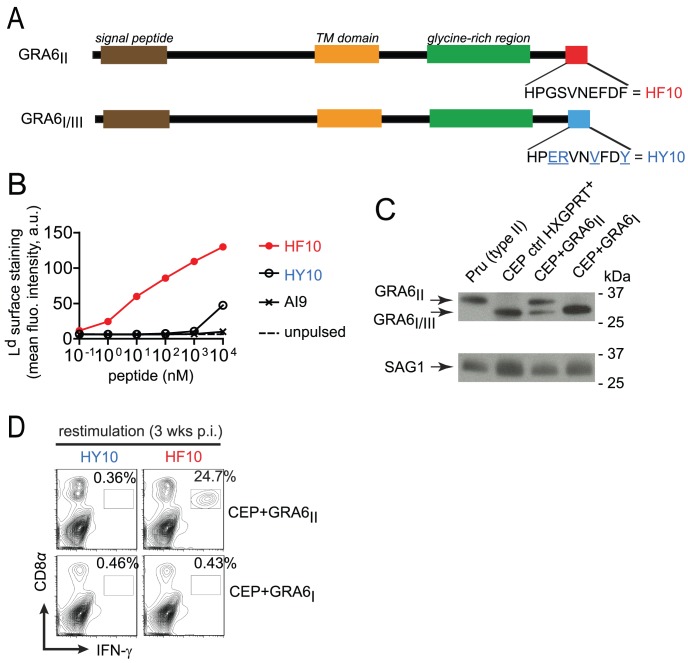
Type III transgenic *T. gondii* to study parasite immunogenicity. (A) Schematics of GRA6 protein from type I/III and type II *T. gondii*. C-terminal amino acid sequence is shown. (B) Surface labeling of peptide-loaded L^d^ analyzed by flow cytometry on TAP-deficient RMA-S.L^d^ cells left unpulsed or pulsed with increasing concentrations of HF10, HY10 or a control D^d^-restricted AI9 peptide. Shown is the mean fluorescence intensity (MFI) in arbitrary units (a.u.). Data representative of 2 independent assays. (C) Western blot analysis of GRA6 (upper panel) and SAG1 (lower panel) in type II Prugnaud (Pru), control CEP (a resistant HXGPRT+ clone which did not integrate the transgene), CEP+GRA6_II_ and CEP+GRA6_I_ clones. Data representative of 4 independent experiments. (D) *Ex vivo* IFN-γ intracellular staining of splenocytes from B10.D2 mice 3 weeks post-infection with CEP+GRA6_II_ (upper plots) or CEP+GRA6_I_ (lower plots), restimulated *in vitro* with HY10 or HF10. Numbers represent the percentage of IFN-γ^+^ out of CD8^+^ cells. Plots show one representative mouse out of 3 per group.

In order to study the pathophysiological consequences of HF10 immunodominance, we generated parasites that do or do not express the GRA6-derived HF10 epitope. We took advantage of the genetic diversity among three common *T. gondii* strains (type I, II and III). While the GRA4-derived SM9 and ROP7-derived IF9 peptides are conserved (data not shown and ToxoDB.org), the GRA6-derived HF10 peptide is polymorphic between type II and type I/III strains. Within the last 10 residues of GRA6, four non-synonymous single-nucleotide polymorphisms differentiate the type II sequence (HF10: HPGSVNEFDF) from the type I/III sequence (HY10: HPERVNVFDY) (Fig. 1A and Fig. S1 in Text S1). We noted that instead of a phenylalanine (F), the C-terminal residue in GRA6_III_ is a tyrosine (Y), a residue not expected to be an appropriate anchor residue for L^d^ binding [21]. To evaluate the ability of HF10 and HY10 to bind to L^d^, we used an MHC I stabilization assay. TAP-deficient RMA-S cells display empty, unstable MHC I molecules and addition of exogenous peptides that can bind to MHC I can stabilize their expression on the cell surface, as read out by flow cytometry. Expression of L^d^ on the surface was stabilized by addition of HF10 at a 1000-fold lower concentration compared to HY10 (Fig. 1B), which confirmed its poor L^d^ binding capacity.

Given that a type III strain like CEP, expresses HY10 (and not HF10), we inferred that it would provide a useful “HF10-null” background to analyze immunodominance *in vivo*. Therefore we engineered CEP parasites to stably express type II or (as a control) type I versions of GRA6 (designated CEP+GRA6_II_ and CEP+GRA6_I_ respectively). To facilitate tracking of parasites and infected cells, we used a CEP strain previously modified to express the GFP and the luciferase reporter genes [22]. We assessed the amount of transgenic GRA6 protein expressed by CEP+GRA6_II_ and CEP+GRA6_I_ parasites by immunoblot, using an antibody that detects all forms of GRA6 (I, II and III). The slower migration of GRA6_II_ allowed us to discriminate between endogenous GRA6_III_ and transgenic GRA6_II_. We confirmed expression of GRA6_II_, at slightly higher levels as compared to endogenous GRA6_III_ in the same parasites. Transgenic GRA6_I_ and endogenous GRA6_III_ were undistinguishable thus precluding a precise analysis of the GRA6_I_ transgene level. (Fig. 1C). Next, we infected B10.D2 mice (H2^d^ MHC haplotype) with CEP+GRA6_II_ and CEP+GRA6_I_ parasites and 3 weeks post-infection, we measured the CD8 T cell response induced by HF10 and HY10 peptides. As observed with the type II Pru strain [17], nearly 25% of CD8 T cells from CEP+GRA6_II_-infected spleens produced IFN-γ in response to HF10 peptide. In contrast, no response was detected above background in CD8 T cells from CEP+GRA6_I_-infected mice after restimulation with the HF10 or HY10 peptides (Fig. 1D). We conclude that CEP strains are “HF10-null” and suitable for assessing the immunogenicity of various transgenes.

### Strong immunodominance of HF10 response but no immunodomination over IF9 and SM9 responses

**Figure 2:**
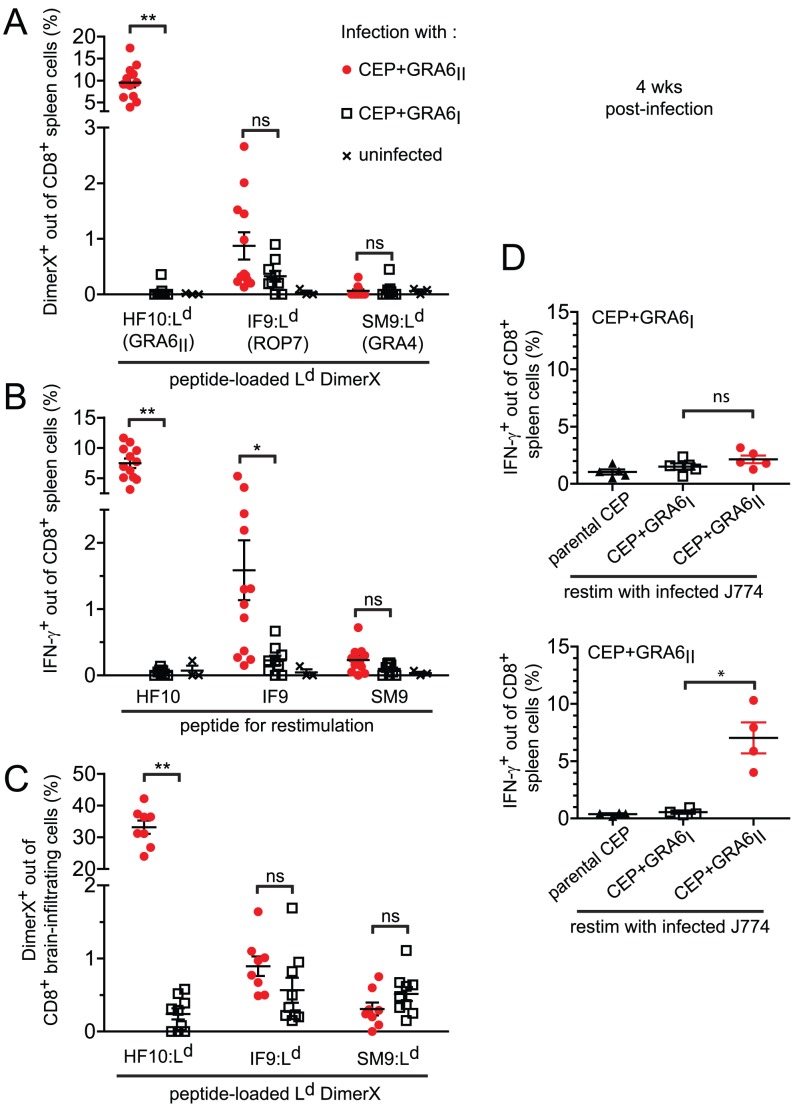
Immunodominance, but not immunodomination, of the GRA6_II_-derived HF10 peptide during chronic stage. For all panels, B10.D2 mice were infected with the indicated parasite strains and analyzed 4 weeks post-infection. (A) Spleen cells stained *ex vivo* with the L^d^ DimerX loaded with the GRA6_II_-derived HF10, ROP7-derived IF9 or GRA4-derived SM9 peptide. Each symbol represents one mouse, horizontal lines represent the mean +/− SEM. Data pooled from 3 independent experiments. (B) *Ex vivo* IFN-γ intracellular staining of spleen cells restimulated with the HF10, IF9 or SM9 peptide. Bars represent the mean +/− SEM. Data pooled from 3 independent experiments. (C) *Ex vivo* L^d^ DimerX staining of brain-infiltrating leukocytes. Bars represent the mean +/− SEM. Data pooled from 2 independent experiments. (D) *Ex vivo* IFN-γ intracellular staining of spleen cells restimulated with J774 macrophages infected *in vitro* with the indicated parasites. Upper panel: mice infected with CEP+GRA6_I_, lower panel: mice infected with CEP+GRA6_II_. Bars represent the mean +/− SEM. Data representative from 2 independent experiments. ns: *P*>0.05 ; *: *P*<0.05 ; **: *P*<0.005.

We used these transgenic parasites to confirm the immunodominance hierarchy among the 3 known natural peptides presented by L^d^ and to analyze the consequences of HF10 absence on T cell responses to the other antigens. Four weeks post-infection, we examined the *T. gondii*-specific CD8 T cell response in the spleen using peptide-loaded L^d^ dimers (Fig. 2A). As expected, a large fraction of HF10-specific CD8 T cells were detected only in CEP+GRA6_II_-infected mice (9.5%+/−3.7%, mean +/− s.d., p = 10^−4^). The IF9-specific CD8 T cells were found at a much lower frequency (0.9+/−0.8%, p = 0.11) and SM9-specific CD8 T cells were hardly detectable. Interestingly, even in the absence of HF10 (such as in CEP+GRA6_I_-infected mice), the frequencies of IF9- and SM9-specific CD8 T cells did not increase, suggesting that the subdominant status of IF9 and SM9 was not the result of competition (also called immunodomination) exerted by HF10-specific T cells. Similar results were obtained when we assessed IFN-γ production by effector CD8 T cells following *in vitro* restimulation (Fig. 2B). Likewise, the epitope-specificity of CD8 T cells in brain infiltrates showed the same HF10>IF9>SM9 hierarchy (Fig. 2C).

While the above experiments define the immunodominance hierarchy among already known epitopes, unknown epitopes could also play a role in the parasite-specific response. To analyze the entire repertoire of *T. gondii*-specific CD8 T cells, we used parasite-infected, rather than peptide-pulsed, APCs to restimulate T cells *ex vivo*. The magnitude of IFN-γ response elicited by parasite-infected macrophages (Fig. 2D) was no higher than that observed after peptide restimulation (see Fig. 2B). Thus, CD8 T cells of other specificities do not play a major role in our experimental model system.

Taken together, our data demonstrate that the presence of GRA6_II_ in the parasites triggers a strong and dominant HF10-specific CD8 response in the spleen and brain of chronically infected animals but does not negatively affect (“immunodominate”) the subdominant SM9 and IF9 responses.

### Expression of HF10 epitope results in diminished parasite burden

**Figure 3:**
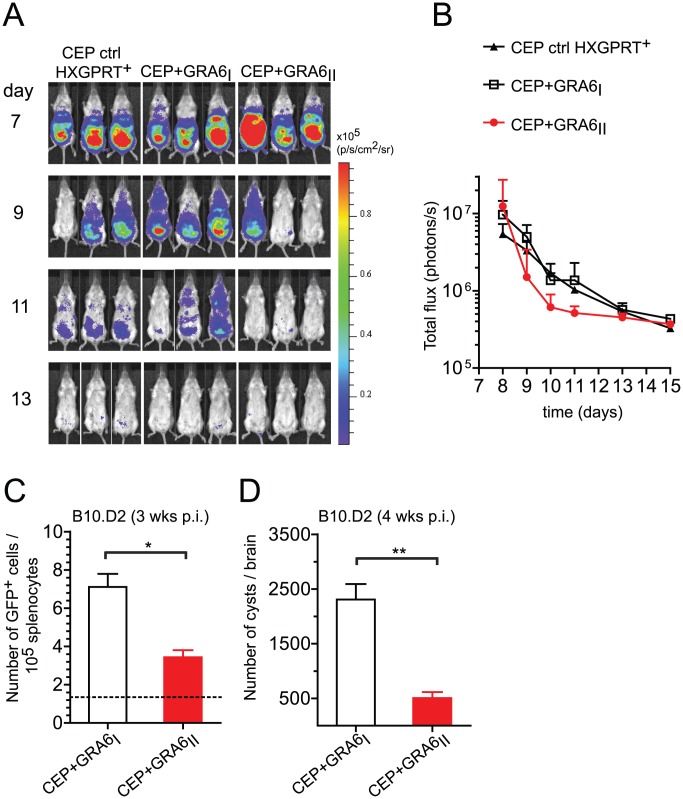
Presence of the GRA6_II_-derived HF10 peptide correlates with better parasite control during acute and chronic *T. gondii* infection. (A,B) BALB/c mice were infected intraperitoneally with 10^5^ tachyzoites of the indicated parasite strains. Parasite clearance was followed from day 7 to 13 by bioluminescence imaging on the ventral side. (A) Raw images of luminescence in pseudocolor scale. (B) Quantification of total flux (photons/s) over time. Dots show the mean + s.d. with 3 mice per group. The differences between CEP+GRA6_I_ and CEP+GRA6_II_ did not achieve statistical significance. Data representative of 2 independent experiments with at least 3 mice per condition. (C,D) B10.D2 mice were infected intraperitoneally with 10^5^ tachyzoites of the indicated parasite strains. (C) Parasite load in the spleen 3 weeks post-infection, as measured by the number of cells harboring a GFP^+^ parasite. Bars show the mean +/− SEM for at least 4 mice per group. The dotted line shows the background in uninfected mice. Data pooled from 2 independent experiments. (D) Parasite burden in the brains of B10.D2 mice 4 weeks post-infection, evaluated microscopically by enumerating the cysts. Histograms represent the mean +/− SEM of at least 5 mice per group. Data pooled from 3 independent experiments. ns: *P*>0.05 ; *: *P*<0.05 ; **: *P*<0.005.

We have previously reported that immunization of H2^d^ mice with HF10-loaded bone marrow-derived dendritic cells protects against lethal type II parasite challenge [17]. We predicted that presence of HF10 may decrease parasite burden. To test this hypothesis, we took advantage of the luciferase expression to analyze parasite dissemination by bioluminescence imaging in BALB/c mice (H2^d^). Regardless of the presence of HF10, all strains were cleared by day 13 (Fig. 3A,B). CEP+GRA6_II_ parasites appeared to be cleared slightly earlier than control CEP (HXGPRT^+^) and CEP+GRA6_I_ parasites, although this difference did not reach statistical significance (Fig. 3A,B). In addition, parasite signal in the brain was detected only in mice infected with control CEP or CEP+GRA6_I_ and was never observed with CEP+GRA6_II_ (Fig. S2 in Text S1). This suggested that the control of parasitemia by HF10-specific T cells may be more effective at the chronic stage, when parasites are found mostly as brain cysts. When we measured parasitemia in chronically infected B10.D2 mice at 4 weeks post-infection, we found a significantly higher proportion of splenocytes harboring parasites in CEP+GRA6_I_-infected mice (Fig. 3C). Accordingly, the number of brain cysts in CEP+GRA6_I_-infected mice was nearly 5 times higher than in mice infected with the HF10-expressing parasites (Fig. 3D). These results could not be attributed to an intrinsic growth difference between clones since they behaved comparably in a plaque assay *in vitro* (Fig. S3A in Text S1). Furthermore, the influence of HF10 on cyst number was visible only in B10.D2 mice and not in C57BL/6 mice, which have a different MHC haplotype (H2^b^) and therefore do not elicit HF10-specific T cells (Fig. S3B in Text S1). Combined, the data show that the HF10-specific response has a modest protective effect on parasite control during acute toxoplasmosis but is essential for controlling parasite load during chronic infection.

### Peptide affinity for L^d^ and naïve T cell frequency cannot explain HF10 immunodominance

**Figure 4:**
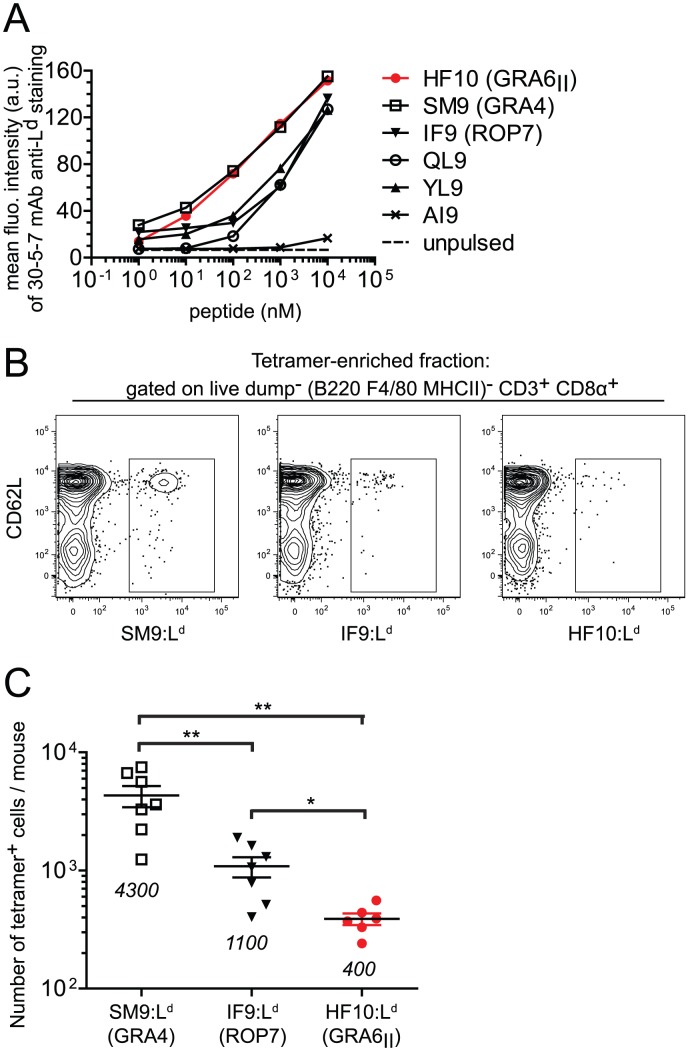
Immunodominance of GRA6_II_-derived HF10 cannot be explained by peptide affinity for L^d^ or naïve T cell frequency. (A) Flow cytometry surface labeling (MFI) of L^d^ on TAP-deficient RMA-S.L^d^ cells left unpulsed or pulsed with increasing concentrations of the indicated L^d^-restricted peptides or a control D^d^-restricted AI9 peptide. Data representative of 3 independent assays. (B,C) Estimation of frequencies of naïve *T. gondii*-specific CD8α^+^ T cell populations in naive B10.D2 mice. (B) Representative flow plots of T cells isolated from spleen and lymph nodes of uninfected mice. Shown are CD62L and tetramer (SM9:L^d^, IF9:L^d^ or HF10:L^d^) stainings after gating on live dump^−^ (dump = B220, F4/80, MHC II) CD3^+^ CD8α^+^ T cells. One million events were collected for each sample. (C) Summary of total tetramer^+^ T cells per mouse. Each dot represents one mouse. Pooled data from 3 independent experiments. Mean +/− SEM are represented by horizontal lines and values are noted on the graph. *: *P*<0.05 ; **: *P*<0.005.

To uncover possible causes of HF10 immunodominance, we used the MHC I stabilization assay described above (see Fig. 1B) and compared the affinity for L^d^ of HF10 to other L^d^-restricted peptides. These other peptides were derived either from *T. gondii* (SM9, IF9), from a mouse minor antigen (QL9) or from a mouse cytomegalovirus protein (YL9) (Fig. 4A). HF10 affinity appeared ∼10-fold higher than that of IF9, QL9 and YL9 but fell in the same range as the *T. gondii* subdominant peptide SM9. Therefore, the dominance hierarchy did not correlate with peptide affinity for MHC I.

We next assessed whether abundance of peptide-specific T cells in the repertoire of naïve mice may control immunodominance. We employed a tetramer-based enrichment procedure [23], [24] to enumerate naïve T cells isolated from spleen and lymph nodes of uninfected mice and specific for each of the 3 epitopes. Numbers of T cell precursors were analyzed by flow cytometry after gating on a population of live dump^−^ (dump = B220, F4/80, MHC II) CD3^+^ CD8α^+^ cells (Fig. S4 in Text S1). Surprisingly, we observed an inverse correlation between the number of naïve T cells per mouse and the immunodominance (Fig. 4B), with the frequency of HF10-specific CD8 T cells around 10- and 3-fold lower than SM9- and IF9-specific CD8 T cells respectively (Fig. 4C). Thus the immunodominance of HF10 cannot be explained by a high starting precursor frequency of specific T cells.

### Location of HF10 at GRA6 C-terminus determines presentation efficiency and parasite control

Having ruled out two plausible hypotheses, we wondered whether HF10 immunodominance might be related to processing efficiency. We noted that HF10 is located at the very C-terminus of GRA6_II_, a position that may facilitate processing since no C-terminal cut is required. To test the importance of epitope position, we changed the C-terminus of HF10 by extending GRA6_II_ with one or more amino acids.

**Figure 5:**
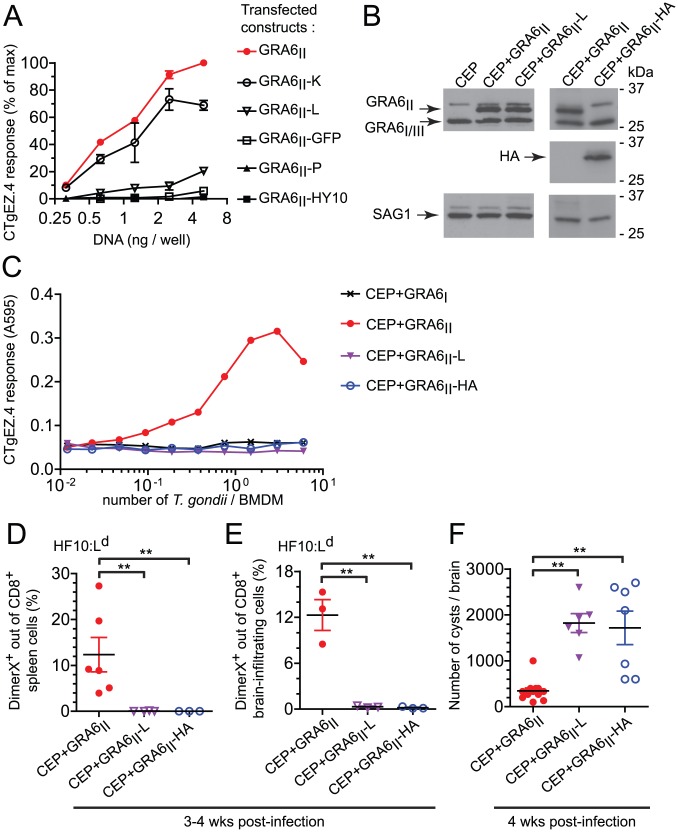
Optimal presentation and parasite control depend on the location of HF10 at the C-terminus of GRA6. (A) HF10 presentation assessed with the CTgEZ.4 hybridomas on mouse fibroblasts co-transfected with L^d^ and plasmids coding for the indicated C-terminally-extended GRA6_II_. Graph shows percentages of maximal response, obtained with 5 ng of wild-type GRA6_II_ plasmid/well. Data are averaged from 2 independent assays +/− SEM. (B) Western blot analysis of GRA6 (upper panels), HA (middle panels) and SAG1 (loading control, lower panels) of the indicated parasites. (C) HF10 presentation by BMDMs infected for 24 h with the indicated parasites, assessed with the CTgEZ.4 hybridomas. Representative of 4 independent experiments. (D,E) *Ex vivo* HF10:L^d^ DimerX staining of spleen cells (D) and brain-infiltrating leukocytes (E) from 3–4 week-infected B10.D2 mice. Each dot represents one mouse, bars show the mean +/− SEM. Data pooled from 2 independent experiments. (F) Cyst burden in the brains of B10.D2 mice infected for 4 weeks. Data pooled from 3 independent experiments. **: *P*<0.005.

We first transfected C-terminally extended versions of GRA6_II_ in mouse fibroblasts. Extensions were either single amino acids (lysine, K ; leucine, L ; proline, P) or several residues such as the GRA6_I/III_-derived HY10 peptide or the entire GFP. We used CTgEZ.4 T cell hybridomas, a β-galactosidase-inducible reporter cell line [17], to read-out HF10 presentation. The response of CTgEZ.4 T cells was mildly decreased (GRA6_II_-K), severely disrupted (GRA6_II_-L) or totally abrogated (GRA6_II_-P, GRA6_II_-HY10, GRA6_II_-GFP) (Fig. 5A). These data suggest that the C-terminal location determines optimal processing and presentation of the HF10 peptide when the precursor protein is expressed ectopically by the antigen-presenting cell.

To assess the impact of HF10 position in *T. gondii*, we used CEP parasites expressing longer versions of GRA6_II_, extended either by a leucine (GRA6_II_-L) or by the HA tag (GRA6_II_-HA). First, we verified that transgene levels were comparable by Western blot (Fig. 5B). To investigate whether these additional C-terminal residues might perturb GRA6 transport, we took advantage of the HA tag and evaluated the subcellular distribution of transgenic GRA6-HA, as compared to total GRA6. Analysis of the overlap between HA and the GRA2 and GRA5 dense granule proteins in extracellular tachyzoites (Fig. S5A,B in Text S1) and in infected fibroblasts (Fig. S5C,D in Text S1), indicated that GRA6_II_-HA is packaged in the dense granules and secreted in the vacuolar space, as known for wild-type GRA6 [25]. Although the distribution of GRA6_II_-L could not be directly assessed, we inferred from the above data that the extra leucine did not alter protein transport either. When used to infect bone marrow-derived macrophages (BMDMs), the CEP+GRA6_II_-L and CEP+GRA6_II_-HA transgenic parasites led to similar infection rates (data not shown) but HF10 presentation was abrogated (Fig. 5C).

Finally, we examined the importance of HF10 C-terminal location *in vivo*. We infected mice and analyzed the induction of HF10-specific CD8 T cells in the spleen (Fig. 5D) and the brain (Fig. 5E) at chronic stage. In accordance with our *in vitro* findings, only the CEP+GRA6_II_ parasites elicited a detectable HF10-specific response. The absence of HF10-specific response in mice infected by CEP+GRA6_II_-L and CEP+GRA6_II_-HA was consistent with a dramatically higher cyst burden in their brains (Fig. 5F). We conclude that the precise C-terminal location of HF10 is required for optimal processing and presentation by *T. gondii*-infected APCs and for eliciting parasite-specific T cells that could provide *in vivo* protection.

### Appending the SM9 peptide to the GRA6 C-terminus overturns its subdominant status

**Figure 6:**
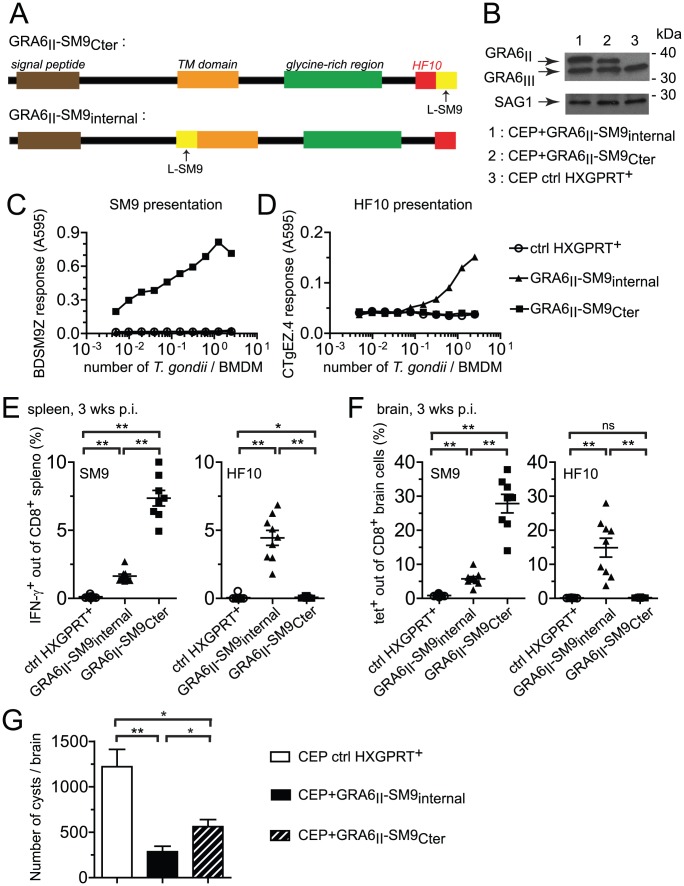
Grafting subdominant SM9 at GRA6_II_ C-terminus enhances its presentation, overturns the dominance hierarchy and provides efficient cyst control. (A) Schematic description of the GRA6_II_-SM9 chimeric constructs. For GRA6_II_-SM9_Cter_, SM9 was introduced at the C-terminus, downstream of HF10. For GRA6_II_-SM9_internal_, SM9 was introduced internally within GRA6_II_ after residue 153, before the putative transmembrane domain. In both cases, SM9 was preceded by a leucine to mimic the endogenous flanking sequence of HF10. (B) Western blot analysis of GRA6 (upper panels) and SAG1 (loading control, lower panels) of the indicated parasite clones. (C,D) SM9 and HF10 presentation by BMDMs infected for 24 h with the indicated parasites, assessed with the BDSM9Z (C) and CTgEZ.4 (D) hybridomas, respectively. (E,F,G) *Ex vivo* evaluation of the CD8 responses and the parasite load 3 weeks post-infection with the indicated parasites. (E) IFN-γ intracellular staining of spleen cells restimulated with the SM9 peptide (left panel) or the HF10 peptide (right panel). (F) Tetramer staining of brain cells with SM9:L^d^ (left panel) and HF10:L^d^ (right panel). Bars represent the mean +/− SEM. Data pooled from 2 independent experiments. (G) Parasite burden in the brains of mice infected with control CEP (white bar), CEP+GRA6_II_-SM9_internal_ (black bar) or CEP+GRA6_II_-SM9_Cter_ (hatched bar), evaluated microscopically by enumerating the cysts. Histograms represent the mean +/− SEM of 14 mice per group, pooled from 3 independent experiments. *: *P*<0.05 ; **: *P*<0.005.

We further assessed whether the location of an epitope at the GRA6_II_ C-terminus may be sufficient for enhancing presentation and immunogenicity. We generated CEP parasites expressing the subdominant SM9 peptide either at the C-terminus of GRA6_II_ (CEP+GRA6_II_-SM9_Cter_) or, as a control, internally within GRA6_II_ (CEP+GRA6_II_-SM9_internal_) (Fig. 6A). The selected clones expressed comparable levels of transgenes (Fig. 6B). We measured SM9 presentation at the surface of parasite-infected cells using a new β-galactosidase-inducible T cell hybridoma specific for L^d^-SM9 complex (BDSM9Z) (Fig. 6C). Interestingly, although the natural SM9 precursor, GRA4, was expressed in type III parasites (data not shown), the presentation of L^d^-SM9 complexes remained below detection in CEP-infected BMDMs. SM9 presentation was also undetectable when the peptide was placed at an internal position within GRA6. In contrast, BDSM9Z T cells were strongly stimulated when SM9 was located at GRA6 C-terminus (Fig. 6C). HF10 presentation by infected BMDMs was abrogated by the presence of SM9 at the C-terminus but not by the presence of SM9 at the internal position (Fig. 6D), consistent the C-terminal extension studies described above (Fig. 5C),. In conclusion, placing the SM9 peptide at the C-terminus of GRA6_II_ was sufficient to enhance its presentation by parasite-infected cells *in vitro*.

We next measured the SM9 and HF10-specific CD8 T cell responses in the spleen (Fig. 6E) and brain (Fig. 6F) of mice infected for 3 weeks with the transgenic parasites. A SM9-specific response was hardly detectable in mice infected with the control CEP. Infection with CEP+GRA6_II_-SM9_internal_ elicited SM9-specific CD8 T cells but these T cells were between 3-fold (spleen, Fig. 6E) and 5-fold (brain, Fig. 6F) more abundant when SM9 was grafted at GRA6 C-terminus. This difference was not due to reduced infectivity of the CEP+GRA6_II_-SM9_internal_ parasites since HF10-specific CD8 T cells were abundant in those mice (Fig. 6E,F). Similar results were obtained in mice immunized with irradiated tachyzoites (Fig. S6 in Text S1). To ask whether the enhanced SM9-specific response participates in parasite control, we enumerated the brain cysts in the 3 groups. As compared to mice infected with control CEP, the parasite load was lower when either a strong HF10- or a strong SM9-specific response was elicited (Fig. 6G). These data indicate that the nature of the antigenic peptide itself does not seem to determine the protective effect. We conclude that location of a subdominant peptide at GRA6 C-terminus dramatically enhanced its immunogenicity, changed the epitope hierarchy and had beneficial repercussions for parasite control.

## Discussion

In this study, we have identified the molecular bases underlying the marked immunodominance of a CD8 T cell response that controls the intracellular *T. gondii* parasite. Rather than peptide affinity for MHC I and naïve T cell frequency, we find that immunodominance is determined by the location of the epitope within the antigenic precursor.

The endeavor to characterize natural T cell antigens from *T. gondii* has started only recently [17], [20], [26], [27] but it has provided much needed tools to better understand T cell immunity to this widespread opportunistic pathogen. We report here that the 3 known L^d^-restricted responses follow an immunodominance hierarchy. At chronic stage, GRA6_II_-specific CD8 T cells were between 10-fold (in the spleen) and 30-fold (in the brain) more abundant than CD8 T cells specific for the 2^nd^ dominant epitope: IF9 derived from ROP7. Response to the 3^rd^ dominant epitope, SM9 derived from GRA4, was hardly detectable. Remarkably, we did not observe immunodomination by the GRA6_II_ dominant epitope. Immunodomination refers to situations in which the T cell response to a given epitope is inhibited by T cells specific for another, more dominant, epitope [2]. This phenomenon has been reported during infection by simian immunodeficiency virus [28] and by *Trypanosoma cruzi*, another protozoan parasite phylogenetically related to *T. gondii* [29]. A mechanism commonly proposed to explain immunodomination is elimination of APCs by the dominant cytotoxic T cells. Perhaps immunodomination does not occur here because, as compared to IFN-γ production, perforin-mediated cytolysis by CD8 T cells plays only a limited role during *T. gondii* infection [30]. The absence of immunodomination also suggests that accessibility of peptide-loaded APCs for T cells is not limiting. This may be because *T. gondii* is able to invade and be presented on MHC I by many cell types, even non-professional APCs [31].

Another major conclusion is that during chronic stage, subdominant responses could not compensate and provide efficient parasite control in the absence of the GRA6_II_ dominant peptide. These data designate GRA6 as a strain-specific component which determines chronic parasitemia and is targeted by adaptive immunity. This is in contrast to already known *T. gondii* virulence factors which mostly interfere with innate processes, such as ROP16 which interferes with STAT transcription factors [32], ROP18 which disarms immunity-related GTPases involved in host defense [33], [34]) or GRA15 which promotes NF-κB activation [35]. Of note, GRA6 is among the 20 most polymorphic genes in the *T. gondii* genome and many polymorphisms are located in its C-terminal region (see ToxoDB.org and Fig. S1 in Text S1). Beyond the 3 prototypic strains, sequence polymorphisms in GRA6 have been characterized in more exotic strains (or haplogroups) [36]. These atypical strains either express HF10, HY10 or alternative versions of the decamer peptide with distinct polymorphisms. Interestingly, in chronically infected humans, some of these variations are specifically recognized by natural antibodies that are used as a tool to serotype the parasite [37], [38]. Given that GRA6 C-terminus is targeted both by humoral and cellular responses, we speculate that selective pressure by adaptive immunity may have contributed in shaping GRA6 polymorphisms.

By exploring the possible causes of HF10 immunodominance, we were able to rule out two possible explanations. We found no positive correlation of immunodominance hierarchy with peptide affinity for L^d^ or naïve T cell frequency. The numbers of naïve HF10- and IF9-specific T cells fall within the previously reported range of 15 to 1500 naïve CD8 T cells per mouse [39] but the number of SM9-specific cells (4300) may look unusually high. Although the exact reason remains unclear, this may be related to a large amount of positive selecting ligands available for this population. In agreement with this idea, an F1 H2^bxd^ mouse strain gives half the value measured with the B10.D2 H-2^d^ strain (data not shown). Remarkably, we found an inverse correlation between size of the naïve population and magnitude of the parasite-specific response. This latter result may seem paradoxical in the light of other situations, such as peptide immunization [23] or viral infection [7], [9], [10], [24], where size of the naïve pool was a good predictor of immunodominance. However, there are known exceptions to this rule [12]. Here, the low abundance of HF10-specific precursors may facilitate their expansion by limiting interclonal competition. Alternatively, the TCRs used by HF10-specific CD8 T cells may have a high affinity for the HF10-L^d^ complex, which could promote stronger signaling and proliferation. These hypotheses remain to be investigated.

Our central finding is that C-terminal position of the epitope within GRA6 plays a crucial role for immunodominance. It is illustrated by the fact that the weakly immunogenic GRA4 epitope elicited strong SM9-specific responses when grafted at GRA6 C-terminus. Intriguingly, the internal position of SM9 gave rise to a lower, but substantial, CD8 response whereas we did not detect any HF10-specific T cells when HF10 was placed at the same internal position (Fig. S7 in Text S1). The bases for such a different outcome are unknown but they may lie in different epitope-dependent processing efficiencies or compensatory effects of the high frequency of SM9-specific T cell precursors counteracting the less favorable position for processing. Most notably, addition of residues to GRA6_II_ C-terminus greatly impaired presentation. It is theoretically possible that the C-terminal flanking sequences abrogated HF10 presentation by altering the vacuolar trafficking of GRA6 and/or its membrane insertion. We think it is unlikely because 1) we found that transport of GRA6_II_-HA was undistinguishable from transport of total GRA6 and 2) presentation of the C-terminal SM9 peptide could occur efficiently *in vitro* and *in vivo*. Rather than regulating global GRA6 trafficking, we favor the idea that additional C-terminal sequences impair processing. Epitope-flanking sequences are indeed known to positively or negatively affect protease cleavage capacity and generation of the final peptide, with clear consequences on immunodominance [4], [6], [40], [41], [42]. Using minigenes, it was shown that the nature of C-terminal flanking residues profoundly impacts excision of the processed peptide [42]. In the context of a full-length viral protein, single changes to the epitope-flanking residues dramatically reduced presentation [41] and it was later proposed that the subdominant nature of certain peptides bearing appropriate consensus motifs might result from suboptimal C-terminal sequences [40]. Finally, it is interesting to note that the influence of the flanking motifs may differ whether the antigen is presented by the direct MHC I pathway or by cross-presentation [43]. In our case, we observed a dramatic impact of the absence or presence of C-terminal residues on presentation, suggesting a key role for antigen processing in modulating immunodominance. A systematic screening of C-terminal extensions may be useful to precisely define the rules that govern processing of GRA6 C-terminus.

Since GRA6 behaves as an integral transmembrane protein in the vacuole [44], the importance of the C-terminus could be related to the topology of GRA6 membrane insertion. One possibility is that GRA6 C-terminal domain is displayed in the cytosol and thus potentially accessible to host proteases. This mechanism was proposed for antigens from the intravacuolar bacteria *Chlamydia trachomatis* that are inserted in the surrounding membrane of the bacteria-containing vacuole [45], [46]. This hypothesis remains to be tested. Alternatively, unfolded GRA6 may access the cytosol thanks to the recruitment of host endoplasmic reticulum components on the parasitophorous vacuole, as proposed for the soluble OVA model antigen [47], [48]. In any case, we consider it likely that access of GRA6 to the MHC I pathway is less efficient than in the situation of a viral antigen directly synthesized by the host cell translation machinery. Consequently, any parameter that would facilitate processing (e.g. being at C-terminus) may become the determining factor for the presentation outcome.

To our knowledge, this is the first evidence that C-terminal position can be positively correlated with immunodominance. Given the variety of parameters that can influence immunodominance, a remaining question is the degree of peculiarity of our current findings with respect to other antigens. A recent study interrogating the Immune Epitope Database (www.iedb.org) for a positional bias of viral epitopes reported that epitopes from both ends of a protein tended to be underrepresented [49]. An indirect way to assess the general relevance of the C-terminal position would be to transfer subdominant epitopes to the C-terminus of their respective antigens (e.g. GRA4, ROP7) and evaluate the impact on CD8 responses. Future studies, not only with *T. gondii* but also with other intracellular parasites, should shed light on the general relevance of this position.

During *T. gondii* infection *in vivo*, two scenarios of MHC I presentation could co-exist. On the one hand, phagocytosed parasite material may be processed by bystander cells present in the vicinity of infected cells [50]. On the other hand, parasite proteins may be directly presented by actively infected cells [51]. Our work shows that antigen access to the MHC I pathway and efficient processing are the limiting factors that control immunodominance. Beyond amino acid mutations within the peptide sequence, modifying the epitope position may provide the parasite with a strategy to manipulate how it is detected by CD8 T cells. Understanding the features that make certain peptides immunogenic will shed light on the strategies used by parasites to interact with their host immune system.

## Materials and Methods

### Ethics statement

In the US, animal studies were carried out in accordance with the recommendations of the Guide for the Care and Use of Laboratory Animals of the National Institutes of Health and in compliance with the guidelines of the Institutional Animal Care and Use Committee (IACUC) of the University of California. The animal protocols were approved by the IACUC of the University of California, Berkeley (Animal Use Protocol # R057-0913BR) and of the University of Pittsburgh, PA (Protocol # 1210130). In France, animal studies were carried out under the control of the National Veterinary Services and in accordance with European regulations (EEC directive 86/609 dated 24 November 1986). The protocol was approved by the Regional Ethics Committee from the Midi-Pyrénées Region (Approval # MP/01/29/09/10).

### Mice and parasites

C57BL/6J (B6), BALB/c and B10.D2-Hc1 H2d H2-T18c/nSnJ (B10.D2) mice were purchased from The Jackson Laboratory (Bar Harbor, ME, USA). B6xDBA/2 F1 (B6D2) mice were purchased from Charles River (France). For all experiments, sex and age-matched mice were used. Mice were handled with the approval of local ethics committees. Except for the CEP+GRA6_II_-HA clone which was a gift from J. Saeij (Cambridge, MA, USA), all transgenic parasites were generated from the parental CEP.ΔHXGPRT.GFP.Luc strain [22]. Tachyzoites were maintained by passage on confluent monolayers of human foreskin fibroblasts (HFF). For infections, parasites were harvested, filtered through 3 µm and 10^5^ tachyzoites were injected intraperitoneally in 100 µl PBS.

### Plasmid constructs, parasite and L cell transfection

For *in vitro* expression of antigenic sequences, all C-terminally extended GRA6_II_ sequences were cloned into the pcDNA1 vector containing the pcDNA1-embedded 3′UTR. Plasmids used for *T. gondii* transfection were derived from the pGRA.HA.HPT vector, a gift from J.D. Dunn and J. Boothroyd (Palo Alto, CA, USA). More details on the construction of the plasmids are given in the Supporting Protocol S1 in Text S1.

L cells were triple transfected using a standard diethylaminoethyl dextran method with vectors coding for L^d^, B7-2 and mutant GRA6, as previously described [17]. For parasite transfections, 1.5×10^7^ tachyzoites were electroporated with 50 µg of HindIII-linearized plasmid DNA and inoculated in 4 confluent HFF flasks in order to obtain up to 4 independent clones. The next day, 25 µg/ml mycophenolic acid and 50 µg/ml xanthine were added for selection. After 2 passages, resistant tachyzoites were cloned by limiting dilution and presence of the transgene was verified by PCR. For each construct, one clone that acquired resistance but no transgene was kept as HXGPRT^+^ control.

### MHC I stabilization assay

RMA-S.L^d^ cells were a gift from T. Hansen (St-Louis, MO, USA). RMA-S.L^d^ cells were incubated at 37°C, 5% CO_2_ for 8 h to saturate the culture medium with CO_2_. The flask was sealed with parafilm and incubated at RT overnight. The next day, cells were washed with PBS and plated at 3×10^5^ cells/well in a 96-W plate. Peptide was added to the cells in serial dilutions. The plate was incubated for 1 h at RT and 3 h at 37°C. Cells were stained with the 30-5-7 antibody (specific for conformed, peptide-bound L^d^) and a phycoerythrine (PE)-coupled goat anti-mouse secondary antibody and analyzed by flow cytometry.

### Western blot

HFFs were disrupted with a 23-G needle and tachyzoites were lysed in a lysis buffer containing 1% NP-40, 10 mM Tris pH 7.4, 150 mM NaCl, and protease inhibitors (cOmplete EDTA-free, Roche) for 30 min on ice. Lysates were centrifuged for 15 min at 15,000 g. Solubilized proteins were boiled and reduced for 5 min in SDS sample buffer, separated by electrophoresis on 12% polyacrylamide gels and transferred to nitrocellulose membranes. Immunologic detection was achieved using rabbit anti-GRA6 serum (gift from L. D. Sibley, St-Louis, MO, USA), mouse anti-HA (gift from D. Raulet, UC Berkeley, CA, USA) or mouse anti-SAG1 (clone TP3, Santa Cruz) followed by secondary horseradish-peroxidase-conjugated antibodies. Peroxidase activity was visualized by chemiluminescence.

### *Ex vivo* cell preparations

Mice were sacrificed 3 to 4 weeks after infection. Spleens were dissociated into single-cell suspensions in complete RPMI (Invitrogen) supplemented with 10% (vol/vol) FCS (Hyclone). Samples were depleted of erythrocytes with ACK lysis buffer (100 µM EDTA, 160 mM NH_4_Cl and 10 mM NaHCO_3_). Leukocytes from the brain were prepared as in [17]. In brief, brains were minced and digested for 1 h at 37°C with 1 mg/ml collagenase (Sigma) and 100 µg/ml DNAseI (Roche) in complete RPMI. Brain suspensions were filtered through 70-µm cell strainers and centrifuged for 10 min at 200 g. Cells were resuspended in 60% (vol/vol) Percoll (GE Healthcare), overlaid on 30% (vol/vol) Percoll and centrifuged 20 min at 1,000 g. Infiltrating mononuclear cells were collected from the gradient interface and the remaining erythrocytes were lyzed with ACK lysis buffer.

### Parasite load analysis

The number of infected splenocytes was determined by measuring the percentage of GFP^+^ cells by flow cytometry. Results from two samples with over 2×10^5^ events collected per tube were averaged for each mouse. For cyst enumeration, the brain was homogenized over a 100 µm strainer and 5% of the entire brain was stained with fluorescein-conjugated *Dolichos biflorus* agglutinin (Vector Laboratories). Cysts were counted using an inverted fluorescence microscope. For bioluminescence imaging, BALB/c mice were infected intraperitoneally with 10^5^ tachyzoites of CEP expressing GRA6_I_ or GRA6_II_ or control CEP HXGPRT^+^. Parasite burden was assessed using *in vivo* bioluminescence imaging as described previously [52]. Briefly, daily readings were performed using an IVIS Lumina II imaging system (Caliper). Ten minutes prior to imaging, mice were injected intraperitoneally with 200 µL of 15.4 mg/mL D-Luciferin in PBS and anesthetized using 2% isoflurane. Dorsal and ventral images were acquired for 5 minutes and luminescence (photons/s/cm^2^/sr, total flux expressed as photons/s) was quantified using IgorPro Image Analysis Software (Caliper).

### Primary macrophage differentiation

Bone marrow cells were obtained from mouse femurs and tibias. Primary BMDMs were differentiated for 7 days in Petri dishes with RPMI supplemented with 20% (vol/vol) FCS and 10% (vol/vol) colony-stimulating factor–containing culture supernatant (purity, about 95% CD11b^+^). Colony-stimulating factor-producing 3T3 cells were a gift from R. Vance (UC Berkeley, CA, USA). BMDMs were infected for 24 h with γ-irradiated tachyzoites (120 Gy) at various multiplicities of infection and used in antigen presentation assays. In all experiments, the proportion of infected (GFP^+^) BMDMs was controlled by flow cytometry.

### Generation of T cell hybridomas and antigen presentation assays

B6D2 F1 mice were immunized subcutaneously with 100 µg synthetic SM9 peptide in complete Freund's adjuvant and boosted after 2 weeks. One week later, spleens were harvested and restimulated with 10 nM SM9. Recombinant human IL-2 (50 U/ml) and 5% T-stim (both from BD Pharmingen) were added after day 2 to support CD8 T cell proliferation. Four days after restimulation, responding cells were fused to the TCRαβ-negative lacZ-inducible BWZ.36/CD8α fusion partner as described in [17]. Specificity of the resulting BDSM9Z hybridomas was tested by overnight incubation with peptide-pulsed or unpulsed L^d^-transfected L cells. TCR-mediated stimulation of the BDSM9Z and the CTgEZ.4 hybridomas [17] was quantified using a chromogenic substrate: chlorophenol red-β-D-galactopyranoside (CPRG, Roche). Cleavage of the CPRG by β-galactosidase releases a purple product, which absorbance was read at 595 nm with a reference at 655 nm.

### Naïve T cell enumeration and flow cytometry

Spleen and major lymph nodes from individual naïve B10.D2 mice were harvested. Single cell suspension was stained with PE-labeled (Prozyme) SM9:L^d^, IF9:L^d^ or HF10:L^d^ tetramers (NIH tetramer facility). Tetramer enrichment was performed on each sample with anti-PE magnetic beads (Miltenyi Biotec) and each sample was stained with antibodies (BD Biosciences) for flow cytometry analysis. Total numbers of CD8α^+^tetramer^+^ T cells per mouse were determined as before [23], [24].

For other stainings, surfaces were labeled according to standard procedures with flow cytometry buffer (3% (vol/vol) FCS and 1 mM EDTA in PBS). Intracellular IFN-γ was detected with the Cytofix/Cytoperm kit (BD Pharmingen). DimerX H-2L^d^:Ig (fusion protein of H-2L^d^ and immunoglobulin; BD Biosciences) was used according to the manufacturer's instructions and as described in [17]. All flow cytometry data were acquired on an XL Analyzer (Coulter) or a LSRII (Becton Dickinson) and were analyzed with FlowJo software (Tree Star).

### Statistical analysis

Prism software (GraphPad) was used for statistical analyses. All *P* values were calculated with the two-tailed Mann-Whitney test (nonparametric).

## Supporting Information

Text S1 Text S1 contains the Supporting Figures S1 to S7 and their respective legends, the: experimental procedures used for the generation of plasmid constructs, the mouse immunizations and immunofluorescence as Supporting protocol S1 and the references cited in the Supporting Figure legends as Supporting References S1.
(DOCX)
