# Supplementary material for: Location of the CD8 T Cell Epitope within the Antigenic Precursor Determines Immunogenicity and Protection against the Toxoplasma gondii Parasite
Source: PLoS Pathog. 2013 Jun 20;9(6):e1003449. doi: 10.1371/journal.ppat.1003449 (PMC3688528; doi:10.1371/journal.ppat.1003449)
Supplement: Text S1 — contains the Supporting Figures S1 to S7 and their respective legends, the: experimental procedures used for the generation of plasmid constructs, the mouse immunizations and immunofluorescence as Supporting protocol S1 and the references cited in the Supporting Figure legends as Supporting References S1. (DOCX) [file ppat.1003449.s001.docx]

**Feliu et al - Supporting Information (Text S1)**

10 20 30 40 50

....|....|....|....|....|....|....|....|....|....|

GT1 (type I) **MAHGGIHLRQKRNFCPVTVSTVAVVFVVFMGVLVNSLGGVAVAADSGGVK**

Me49 (type II) **......Y.........L................................R**

VEG (type III) **................L.............................D...**

60 70 80 90 100

....|....|....|....|....|....|....|....|....|....|

GT1 (type I) **QTPSETGSSGGQQEAVGTTEDYVNSSAMGGGQGDSLAEDDTTSEAAEGDV**

Me49 (type II) **...........................................D......**

VEG (type III) **...........................................D......**

110 120 130 140 150

....|....|....|....|....|....|....|....|....|....|

GT1 (type I) **DPFPVLANEGKSEARGPSLEERIEEQGTRRRYSSVQEPQAKVPSKRTQKR**

Me49 (type II) **....A.............................................**

VEG (type III) **..................................................**

160 170 180 190 200

....|....|....|....|....|....|....|....|....|....|

GT1 (type I) **HRLIGAVVLAVSVAMLTAFFLRRTGRRSPQEPSGDGGGNDAGNNAGNGGN**

Me49 (type II) **..................................G...............**

VEG (type III) **.............................P.................R..**

210 220 230

....|....|....|....|....|....|

GT1 (type I) **EGRGYGGRGEGGAEDDRRPLHPERVNVFDY**

Me49 (type II) **...-----....-.........GS..E..F**

VEG (type III) **............G.....A...........**

**Figure S1: Amino acid sequence alignment of GRA6_I_, GRA6_II_ and GRA6_III_ (related to Figure 1)**

Sequences of the 3 genotypes were retrieved from ToxoDB.org and aligned using the BioEdit software and the ClustalW algorithm. Dots indicate identity with respect to the type I GRA6 reference sequence. Dashes indicate missing residues. Note that in the C-terminal domain, 6 residues are missing in the type II sequence (total length 224 aa *vs.* 230 aa for type I/III). Regions of interest are highlighted and color-coded like in the main figures. Brown: signal peptide ; orange: TM domain ; green: glycine-rich region ; blue: HY10 peptide ; red: HF10 peptide.

**Figure S2: No brain parasitemia detected during acute *T. gondii* infection in the presence of the GRA6_II_-derived HF10 peptide (related to Figure 3)**

BALB/c mice were infected intraperitoneally with 10^5^ tachyzoites of the indicated parasite strains. (A) Images show the parasite signal followed from day 11 to 14 by bioluminescence imaging on the dorsal side as previously described in Supporting references S1 [[1](#_ENREF_1),[2](#_ENREF_2),[3](#_ENREF_3)]. (B) Quantification of total flux (photons/s) over time in the brain, integrated over the red ROI. Experiment performed only once.

**Figure S3: Growth of CEP+GRA6_I_ and CEP+GRA6_II_ clones is comparable *in vitro* and *in vivo* in C57BL/6 mice (related to Figure 3)**

(A) Number of Giemsa-negative plaques counted 9 days after inoculation of a 25-cm^2^ flask of confluent HFF with 5x10^3^ of the indicated parasites. Experiment performed only once. (B) Parasite burden in the brains of C57BL/6 mice 4 weeks post-infection, evaluated microscopically by enumerating the cysts. Histograms represent the mean +/- SEM of at least 4 mice per group. Data pooled from 2 independent experiments. ns: *P* > 0.05

**Figure S4: Gating strategy to enumerate naïve CD8α^+^tetramer^+^ T cells from tetramer-enriched fraction (related to Figure 4)**

Cells were isolated from spleen and pooled lymph nodes of uninfected B10.D2 mice, labeled with MHC I tetramers, enriched with a magnetic-based procedure and further stained for flow cytometry. Gating was performed with non-fluorescent parameters to exclude doublets and then on live, CD3^+^ dump^-^ (B220 F4/80 MHC II)^-^ CD8α^+^ or CD4^+^ cells. Levels of the activation marker CD62L and of the tetramer staining were analyzed.

**Figure S5: Subcellular localization of total GRA6 and transgenic GRA6_II_-HA, compared with GRA2 and GRA5**

Representative examples of (A,B) extracellular CEP+GRA6_II_-HA tachyzoites and (C,D) HFF infected for 24h with CEP+GRA6_II_-HA tachyzoites, stained with mouse anti-GRA2 and rabbit anti-GRA6 or anti-HA antibodies (upper panels) or mouse anti-GRA5 and rabbit anti-GRA6 or anti-HA antibodies (lower panels). The anti-GRA6 antibody recognizes both endogenous GRA6_III_ and transgenic GRA6_II_-HA whereas the anti-HA antibody detects only transgenic GRA6_II_-HA.

(A,B) In extracellular parasites, there is excellent colocalization between dense granule markers (GRA2 and GRA5) and total or transgenic GRA6, showing that transgenic GRA6_II_-HA is contained within the dense granules. Pearson’s correlation coefficients (Pcc) > 0.9 for all analyzed tachyzoites.

(C,D) In infected HFF, total GRA6 and transgenic GRA6-HA are found in the vacuolar space in between the parasites (tubulovesicular network) and also partially at the vacuole limiting membrane. GRA6 and HA stainings show a strong overlap with GRA2 (0.93 > Pcc > 0.83) which associates with the tubulovesicular network (Supporting references S1 [[4](#_ENREF_4)]) and a more limited overlap with GRA5 (0.85 > Pcc > 0.7), which is targeted at the vacuole limiting membrane (Supporting reference S1 [[5](#_ENREF_5)]) as pointed by the white arrows.

In conclusion, GRA6_II_-HA appears to be secreted in the vacuole and to accumulate at a location similar to the one known for endogenous GRA6.

**Figure S6: Grafting subdominant SM9 at GRA6_II_ C-terminus enhances its presentation and overturns the dominance hierarchy after immunization with irradiated tachyzoites (related to Fig. 6)**

(A,B) *Ex vivo* IFN-γ intracellular staining of spleen cells (upper panels) and peritoneal cells (lower panels) 7 days following immunization with the indicated parasites. Cells were restimulated either with the SM9 peptide (A) or the HF10 peptide (B). Bars represent the mean +/- SEM. Data pooled from 3 independent experiments. *: *P* < 0.05 ; **: *P* < 0.005

**Figure S7: In contrast to internal SM9, internal position of HF10 does not elicit a detectable HF10-specific response (related to Fig. 6)**

(A) Schematics of the GRA6_II_-SM9 chimeras and the GRA6_II_-HF10_internal_-HY10_Cter_ construct containing the HF10 peptide placed at the same internal location. To avoid truncating the protein and preserve a more « natural » C-terminal amino acid sequence, HF10 was replaced by HY10 (the equivalent non-immunogenic type I sequence) at the C-terminus. In all cases, SM9 and HF10 were preceded by a leucine to mimic the endogenous flanking sequence of HF10. (B) *Ex vivo* MHC I tetramer stainings of spleen cells (upper panels) and brain cells (lower panels) 3 weeks post-infection with the indicated live parasites. Bars represent the mean +/- SEM. Data from one experiment. Similar results were obtained when IFN-γ production was assayed at day 7 following immunization with irradiated parasites.

**Supporting Protocol S1**

**Generation of plasmid constructs**

For *in vitro* expression of antigenic sequences, all C-terminally extended GRA6_II_ sequences were cloned into the pcDNA1 vector containing the pcDNA1-embedded 3’UTR. GRA6_II_-K, GRA6_II_-P, GRA6_II_-L and GRA6_II_-HY10 coding sequences were obtained by PCR using pcDNA1.GRA6_II_ (known as G93 plasmid) as template, a T7 forward primer and reverse primers annealing to the 3’ end of GRA6 (without the stop codon), including the C-terminal extension and a NotI site. PCR amplicons were cloned into pcDNA1 using BstXI and NotI. pcDNA1.GRA6_II_-GFP was obtained 1/ by PCR of GFP ORF using the pEGFP-N3 plasmid (BD Clontech) as template, a forward primer containing the 3’ end of GRA6_II_ ORF from the XmaI site down to the last C-terminal nucleotide (without stop codon) plus the 5’ end of GFP ORF, a reverse primer annealing to the 3’ end of GFP ORF and including a NotI site ; 2/ cloning of the modified GFP amplicon in G93 plasmid downstream of GRA6_II_ using XmaI and NotI.

Plasmids used for *T. gondii* transfection were all derived from the pGRA.HA.HPT vector, a gift from J.D. Dunn and J. Boothroyd (Palo Alto, CA, USA). Wild-type GRA6_I_ and GRA6_II_ coding sequences were amplified by PCR using genomic DNA from type I RH or type II Prugnaud as respective templates, a forward primer annealing ~2 kb upstream of the GRA6 initiation codon and containing a HindIII site and a reverse primer annealing ~500 bp downstream of GRA6 3’ UTR and containing a NotI site. GRA6_I_ and GRA6_II_ amplicons (including the endogenous 5’ and 3’ UTR) were cloned into pGRA.HA.HPT using HindIII and NotI. Plasmids encoding GRA6_II_-L, GRA6_II_-SM9_Cter_ and GRA6_II_-SM9_internal_ were obtained using site-directed mutagenesis on the pGRA.HPT.GRA6_II_ vector. All plasmids were validated by restriction digest and sequencing. Primer sequences are available upon request.

Of note, all GRA6 transgenes (except GRA6-HA) were flanked by the endogenous GRA6_II_ promoter plus 5’UTR sequence and by the endogenous GRA6_II_ 3’ UTR. GRA6-HA expression was driven by the GRA1 promoter plus 5’UTR and followed by the GRA2 3’UTR.

**Mouse immunizations**

Mice were immunized with 2x10^6^ γ-irradiated (120 Gy) parasites previously filtered on 3 µm filters and T cell responses were analyzed 7 days later.

**Immunofluorescence**

HFF monolayers were grown on 8-well LabTek slides (Nunc) and infected with CEP+GRA6_II_-HA tachyzoites for 24h. Alternatively, freshly lyzed extracellular CEP+GRA6_II_-HA tachyzoites were sedimented on LabTek slides. Cells were washed with PBS, fixed for 15 min with 3% PFA (Electron Microscopy Sciences), quenched with PBS 0.1M glycine for 5 min and permeabilized with PBS, BSA 0.2%, saponine (Sigma) 0.05% for 5 min. Primary antibodies and fluorochrome-coupled secondary antibodies were added in the permeabilization buffer for 30 min each at RT. Cells were washed and mounted on a glass slide with Mowiol. Images were acquired with a 63X objective on a Zeiss LSM510 confocal microscope. Mouse antibodies to GRA2 and GRA5 N-terminus were a gift from M.-F. Delauw (Grenoble, France). Rabbit anti-HA was from Sigma. Alexa-Fluor-coupled secondary antibodies were from Molecular Probes.

**Supporting references S1**

1. Hitziger N, Dellacasa I, Albiger B, Barragan A (2005) Dissemination of Toxoplasma gondii to immunoprivileged organs and role of Toll/interleukin-1 receptor signalling for host resistance assessed by in vivo bioluminescence imaging. Cellular microbiology 7: 837-848.

2. Saeij JP, Boyle JP, Grigg ME, Arrizabalaga G, Boothroyd JC (2005) Bioluminescence imaging of Toxoplasma gondii infection in living mice reveals dramatic differences between strains. Infect Immun 73: 695-702.

3. Kim SK, Karasov A, Boothroyd JC (2007) Bradyzoite-specific surface antigen SRS9 plays a role in maintaining Toxoplasma gondii persistence in the brain and in host control of parasite replication in the intestine. Infect Immun 75: 1626-1634.

4. Mercier C, Cesbron-Delauw MF, Sibley LD (1998) The amphipathic alpha helices of the toxoplasma protein GRA2 mediate post-secretory membrane association. J Cell Sci 111: 2171-2180.

5. Gendrin C, Mercier C, Braun L, Musset K, Dubremetz JF, et al. (2008) Toxoplasma gondii uses unusual sorting mechanisms to deliver transmembrane proteins into the host-cell vacuole. Traffic 9: 1665-1680.
